# Rapid, Room-Temperature Synthesis of a Porous Organic Polymer for Highly Effective Removal of Trace Hg(II) from Water

**DOI:** 10.3390/molecules30234635

**Published:** 2025-12-02

**Authors:** Shucai Gao, Libin Wan, Fayun Wang, Haidong Gao, Fanghui Zhao, Na Li, Jingjing Yao, Yeru Liu, Hongwei Liu

**Affiliations:** 1 Institute of Business Scientific, Henan Academy of Sciences, Wenhua Road #87, Zhengzhou 450003, China; gsc@hnas.ac.cn (S.G.); wangfayun262@sohu.com (F.W.); ghd@hnas.ac.cn (H.G.); fhzhao98@163.com (F.Z.); linayu1985@163.com (N.L.); yaojingjing@hnas.ac.cn (J.Y.); liuyeru@hnas.ac.cn (Y.L.); liuhongwei@hnas.ac.cn (H.L.); 2 College of Chemistry and Molecular Engineering, Zhengzhou University, Zhengzhou 450001, China

**Keywords:** covalent organic frameworks, room-temperature, trace level, mercury adsorption

## Abstract

Exposure to Hg^2+^-contaminated water poses severe risks to human health. Porous organic polymers (POPs) are known for removing heavy metals efficiently. However, the rapid and simple preparation of POP with efficient and selective adsorption capacities remains challenging. Herein, an effective strategy for the room-temperature preparation of TpPa-1 via a 1-step Schiff-base reaction of 2,4,6-triformylphloroglucinol (Tp) and p-phenylenediamine (Pa-1) using scandium(III) trifluromethanesulfonate as a catalyst is described. Various approaches were used to characterize TpPa-1, including SEM, TEM, XRD, FT-IR, NMR, BET, and TG analysis. TpPa-1 was applied to adsorb trace Hg^2+^ from aqueous solution, and its adsorption performance was assessed through batch adsorption experiments. The results indicated that over 94% of 100 μg L^−1^ Hg^2+^ was removed within 90 min, with the isotherm and kinetics conforming to the Freundlich and the pseudo-second-order models, respectively. Combined with XPS analysis, the Hg^2+^ adsorption of TpPa-1 was primarily dominated by chelation, competitive, and electrostatic interactions between the carbonyl groups of TpPa-1 and Hg^2+^. Because of its benefits of facile synthesis, enhanced removal performance, good selectivity, and reusability, the prepared POP material has great potential for Hg^2+^ removal from aqueous solutions.

## 1. Introduction

With the fast advancement of global industrialization, wastewater containing various pollutants generated in the industrial production process has been discharged into the water environment, especially heavy metal wastewater. Among heavy metal pollutants, mercury (Hg) is among the most toxic, and also exhibits extensive distribution in the environment [1,2]. The fundamental contributors of Hg are anthropogenic emissions from industrial production, including metal smelters, thermometer factories, and pharmaceutical factories, which eventually enter the water environment [3]. These Hg pollutants in polluted water can easily be transformed into highly toxic methylmercury by microorganisms, and eventually infiltrate the body via the food chain, thereby presenting threats to both human and ecological health [4]. Hg pollution in water is extremely difficult to degrade naturally. It is highly toxic, long-lasting, and accumulates in organisms. Even at very low exposure levels, it may exert strong biological effects, leading to carcinogenic, teratogenic, and mutagenic outcomes [5]. Therefore, developing an inexpensive and effective method for separating trace Hg from water is imperative.

In recent years, numerous methods for removing Hg pollutants from water have been suggested, such as chemical precipitation [6], membrane separation [7], ion exchange [8], biological approach [9], and adsorption [10]. Most of these methods are effective in removing high levels of Hg^2+^, but their removal efficiency of trace Hg^2+^ is unsatisfactory. The residue concentration of Hg^2+^ in effluent after treatment is at the level of μg L^−1^, consistent with the detected concentration of Hg^2+^ in natural water environments [11,12]. The adsorption technique has garnered significant interest among these approaches, owing to its high performance and low cost. It also offers the benefits of minimal secondary pollution and provides high effluent quality [13]. For the adsorption method, the key lies in developing high-capacity adsorbents. However, the efficiency of trace Hg^2+^ removal by traditional adsorbents is poor, primarily because of their limited specific surface area and weak affinity for Hg^2+^ [14,15].

Porous organic polymers (POPs) are porous materials constructed via the covalent bonding of specific elements (e.g., N, O, C, and H), which endow them with a highly specific surface area, facile functional modification, and high chemical and thermal stability. These properties make POPs suitable for a wide range of applications, including gas capture, adsorption, separation, and catalysis [16,17]. Based on their structural features, POPs are mainly divided into crystalline and amorphous types, with covalent organic frameworks (COFs) serving as the representative crystalline form. Previous studies have reported that sulfur-based groups (-SR) exhibit high selectivity for Hg^2+^, primarily linked to the development of strong soft–soft chelation bonds between -SR groups and Hg^2+^ [18,19]. Hence, grafting -SR groups onto the molecular skeleton of COFs either by predesigning organic building blocks modified with -SR groups or via post-synthetic modification has become a common and effective strategy to enhance Hg^2+^ removal efficiency [20]. For example, Huang et al. [21] designed a methylthio-modified organic building block to synthesize TPAB-DMTTPA COF at 120 °C over 3 days, achieving high efficiency for Hg^2+^ capture. Sun et al. [22] developed a COF-S-SH adsorbent by grafting ethanedithiol onto a vinyl-functionalized COF after synthesis. This material exhibited high Hg^2+^ removal efficiency. Additionally, Pan et al. [10] synthesized COF-SH-1 and COF-SH-2 through a Schiff-base reaction by grafting trithiocyanuric acid or bismuththiol onto vinyl-functionalized COF. These materials exhibited high respective Hg^2+^ adsorption efficiencies of 763.4 and 526.3 mg g^−1^. Although sulfur-functionalized COFs generally show higher Hg^2+^ adsorption capacities, their regeneration is difficult because of the strong Hg–S affinity [23]. In addition to -SR groups, other functional groups, including oxygen-rich and nitrogen-rich groups, have also been used to enhance the affinity of Schiff-base materials toward Hg^2+^ [24]. For instance, an azine-modified COF synthesized with 1,3,5-triformylphloroglucinol and hydrazine monomers at 80 °C over 3 days showed enhanced adsorption (177 mg g^−1^, with a removal capability of 70.8%) for Hg^2+^ (starting concentration equal to 100 mg L^−1^) at pH = 12. This performance was ascribed primarily to coordination interactions of Hg^2+^ with C=O and -NH groups in the COF keto form [25]. Li et al. [26] found that high oxygen and nitrogen densities in TpODH-COF contributed to strong Hg^2+^ affinity, resulting in an adsorption efficiency of 1692 mg g^−1^, which is higher in comparison to that of COF-SR materials reported in prior literature.

**Figure 1:**
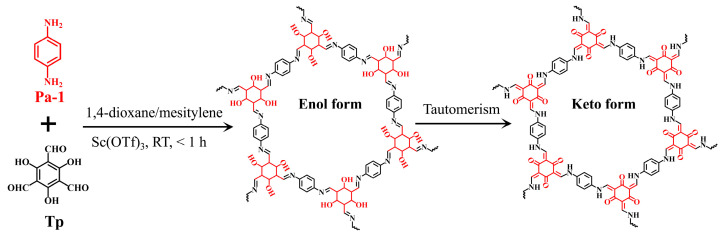
Schematic representation of the synthesis of TpPa-1 material.

To date, various COFs have been synthesized using different methods, including solvothermal [27], microwave-assisted [28], mechanical grinding [29], and room-temperature [30,31] syntheses. However, most of these methods need high temperatures (80–170 °C) and prolonged reaction times (12–72 h), which increase energy costs and operation complexity, thereby limiting their application scope. To address these issues, in the present work, we propose a facile, rapid, and mild method for the room-temperature fabrication of TpPa-1 via a Schiff-base reaction between p-phenylenediamine (Pa-1) and 2,4,6-triformylphloroglucinol (Tp) using scandium(III) trifluromethanesulfonate (Sc(OTf)_3_) as a catalyst (Figure 1). The crystalline structure, surface properties, thermal stability, and morphology of TpPa-1 were thoroughly characterized using various techniques. The adsorption kinetics, isotherm, and selectivity of TpPa-1 for trace Hg^2+^ were investigated to evaluate its adsorption performance. The reusability of TpPa-1 was studied over 5 consecutive desorption/adsorption cycles. Additionally, the effect of pH on Hg^2+^ capture was explored, and XPS characterization was employed to explore the mechanism responsible for adsorption.

## 2. Results and Discussion

### 2.1. Synthesis and Analysis of TpPa-1

**Figure 2:**
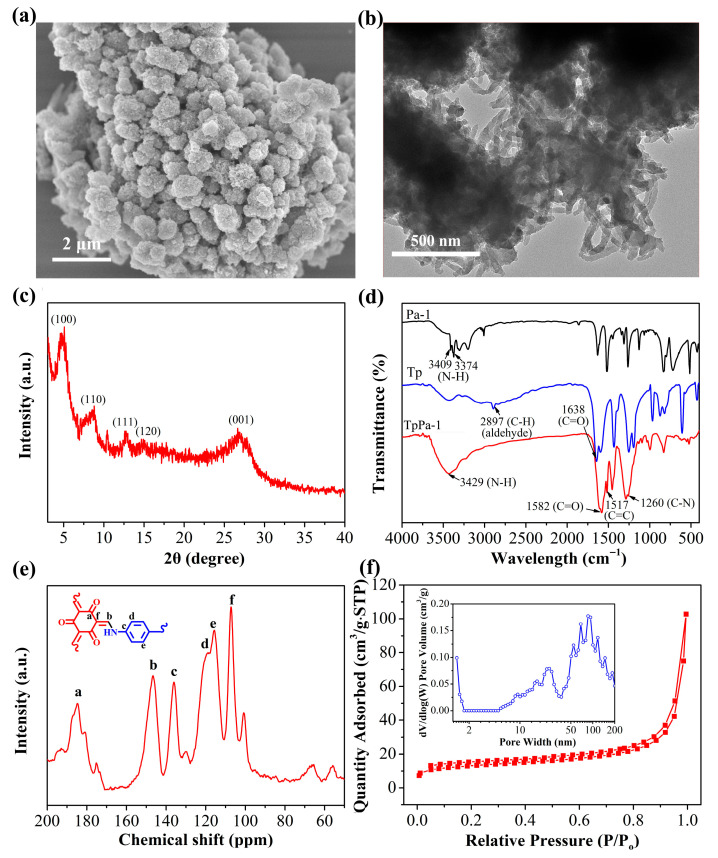
(**a**) SEM and (**b**) TEM micrographs of TpPa-1. (**c**) The XRD pattern obtained for TpPa-1. (**d**) The FT-IR spectra obtained for Tp, Pa-1 and TpPa-1. (**e**) The ^13^C solid NMR spectrum acquired for TpPa-1 (the lowercase letters represent different carbon (C) atoms). (**f**) The N_2_ adsorption–desorption isotherm and DFT pore size distribution of TpPa-1.

The morphology of the synthesized TpPa-1 was characterized via TEM and SEM. The SEM image revealed that TpPa-1 forms cage-like spheres composed of nanorods, with a diameter of approximately 0.8–1.0 μm, a rough surface, and a porous structure, resulting from the stacking and assembly of nanorods (Figure 2a). From the TEM image in Figure 2b, TpPa-1 exhibits a distinct spherical structure assembled from stacked nanorods, consistent with the SEM findings. Figure 2c shows the XRD pattern of TpPa-1. A high-intensity XRD peak was observed at 4.8°, identifying the (100) crystal plane reflection. Additional characteristic peaks are observed at 8.4°, 12.7°, and 14.6°, representing the reflections of the (110), (111), and (120) crystal planes, respectively [32]. Two weak peaks around 26.7° are attributed to π-π stacking between different COF layers. These XRD results indicate the success of the TpPa-1 preparation.

The FT-IR spectra of Pa-1, Tp, and TpPa-1 are illustrated in Figure 2d. In Pa-1, the peaks observed at 3300–3400 cm^−1^ were linked to the N-H stretching vibrations. In the Tp spectrum, the 2897 and 1638 cm^−1^ bands correlate, respectively, with C-H and C=O stretching within the aldehyde moiety. Compared with Tp and Pa-1, the TpPa-1 spectrum exhibits respective stretching vibration peaks for N-H, C=O, C-N, and C=C bonds at 3429, 1582, 1260, and 1517 cm^−1^, respectively [33]. These observations confirm the effective fabrication of TpPa-1 via Schiff-base reactions. ^13^C solid-state NMR spectroscopy was utilized to study the structure of TpPa-1 (Figure 2e). In the ^13^C CP/MAS NMR spectrum obtained for TpPa-1, the characteristic peaks at 184.8 and 146.5 ppm are associated with the C=O (C_a_) and C-N-Ar (C_b_) groups, respectively, while peaks at 136.0, 119.0, 115.7, and 107.2 ppm correspond to C_c_, C_d_, C_e_, and C_f_, respectively [34]. The findings confirm that TpPa-1 was successfully synthesized via Schiff-base reaction, which correlates with the FT-IR findings. The porosity and specific surface area of TpPa-1 were explored via N_2_ adsorption at 77 K (Figure 2f). The N_2_ adsorption isotherm obtained for TpPa-1 exhibited a typical type II behavior. Based on these results, combined with the DFT pore size distribution analysis (the inset figure in Figure 2f), TpPa-1 possessed a mesoporous morphology. The BET specific surface area was found as 47.11 m^2^/g, which is significantly smaller than those reported for similar COF. This discrepancy is likely due to the low crystallinity of the obtained TpPa-1, suggesting that this TpPa-1 material may be a partially amorphous POP material. The thermal stability was tested by TG analysis under an air atmosphere (Figure S1). As shown in the TG curve of the TpPa-1, there is no obvious weight loss below 400 °C. A weight loss of 40% occurs between 400 °C and 500 °C, likely the result of framework decomposition, demonstrating the thermal stability of TpPa-1 to temperatures up to 400 °C. The content of scandium ions from the TpPa-1 after soaking in aqueous solution for 12 h was determined by ICP-MS. The result showed that scandium ions could not be detected, suggesting the safety of TpPa-1 adsorbent.

### 2.2. Adsorption Behavior of TpPa-1

#### 2.2.1. Kinetics

**Figure 3:**
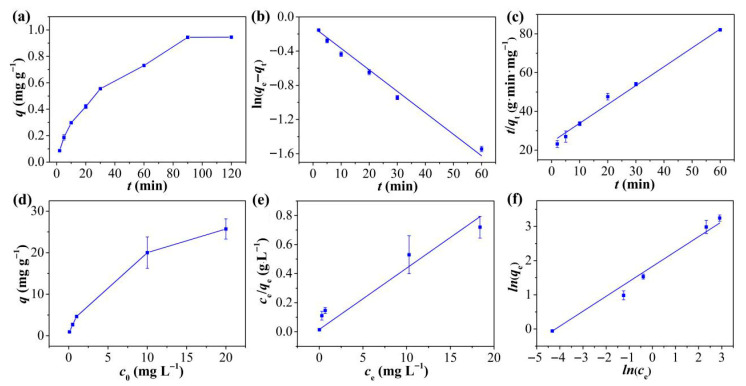
(**a**) Kinetic adsorption curve of Hg^2+^ on TpPa-1. (**b**) PFO and (**c**) PSO kinetics of adsorption for Hg^2+^ on TpPa-1. (**d**) Equilibrium adsorption curve of Hg^2+^ on TpPa-1. (**e**) Langmuir and (**f**) the Freundlich isotherm adsorption models of Hg^2+^ on TpPa-1.

Figure 3a displays the kinetic curves of Hg^2+^ adsorption on TpPa-1 over different time intervals. Hg^2+^ adsorption onto TpPa-1 was rapid within the first 20 min due to the plentiful free adsorption sites available on TpPa-1 initially [35], reaching equilibrium by 90 min. The kinetic behavior was examined by employing PFO and PSO models for fitting the adsorption findings of Hg^2+^ onto TpPa-1. The corresponding fitting curves are depicted in Figure 3b,c, and the fitting conditions are mentioned in Table S1. The adsorption of Hg^2+^ onto TpPa-1 adheres to PSO kinetic behavior (R^2^ = 0.9946) more closely relative to PFO behavior (R^2^ = 0.9877), indicating that Hg^2+^ removal is dominated by chemical adsorption [36]. This is particularly notable for TpPa-1, given its limited specific surface area and porous structure. The calculated equilibrium adsorption capacity (*q_e_*, 1.03 mg g^−1^) closely aligns with the empirically determined value (0.94 mg g^−1^).

#### 2.2.2. Isotherms

The isotherm of Hg^2+^ adsorption on TpPa-1 was explored for assessing the adsorption efficiency of the adsorbent. It is evident from Figure 3d that the initial amount of Hg^2+^ had a marked influence on the overall adsorption ability of TpPa-1. The equilibrium adsorption capacity increased markedly as the starting Hg^2+^ level rose from 0.1 to 20 mg L^−1^, with no saturation observed within this concentration range. This suggests that TpPa-1 possesses abundant active adsorption sites for Hg^2+^. As reported in prior literature [37,38], the Langmuir isotherm typically describes adsorption of monolayers on homogeneous surfaces, whereas the Freundlich model represents either multilayered adsorption or interactions with heterogeneous surfaces. To further characterize the adsorption behavior and surface properties of TpPa-1, the empirical findings were verified via the Freundlich and the Langmuir models. The fitted plots are displayed in Figure 3e,f, and the relevant conditions are mentioned in Table S2. The Freundlich model exhibited a higher correlation coefficient (R^2^ = 0.9924) in comparison to the Langmuir model equation (R^2^ = 0.6985), indicating that the adsorption of Hg^2+^ onto TpPa-1 aligns more closely with the Freundlich description, implying that Hg^2+^ adsorption involves multi-molecular layer interactions on the TpPa-1 surface. Additionally, the calculated 1/*n* value of 0.436 suggests favorable adsorption interactions between Hg^2+^ and TpPa-1.

#### 2.2.3. pH

**Figure 4:**
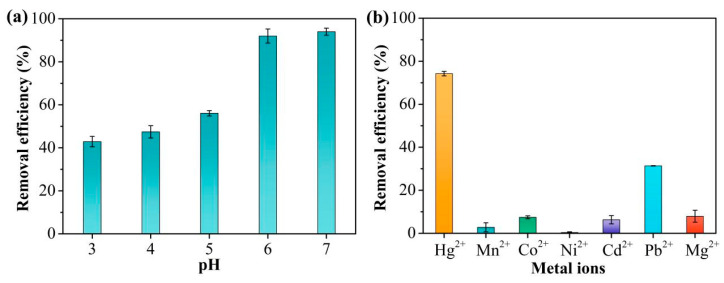
(**a**) Hg^2+^ adsorption to TpPa-1 at different solution pHs. (**b**) The selective adsorption in the presence of metal ions.

The pH can have marked effects on the adsorption characteristics of TpPa-1 for Hg^2+^. Changes in the pH influence both the surface charge of TpPa-1 and the existing forms of Hg^2+^. The adsorption efficiency of TpPa-1 for Hg^2+^ at different solution pH values is illustrated in Figure 4a. As the pH rose from 3 to 7, the effectiveness of Hg^2+^ removal improved. At pH = 7, the Hg^2+^ adsorption rate was the highest (over 90%), which was close to that at pH = 6. Combined with the zeta potential analysis of TpP-1 (Figure S2), it may be because when the pH was less than 6, the increased number of H^+^ ions led to protonation of TpPa-1, thus resulting in a low adsorption capacity of Hg^2+^ [39].

### 2.3. Adsorption Selectivity

In general, there are a large number of other coexisting ions in Hg^2+^-contaminated water. Therefore, the ability to selectively adsorb Hg^2+^ from water is crucial. To assess the influence of coexisting ions, Hg^2+^ adsorption to TpPa-1 was evaluated when other ions, namely, Mn^2+^, Co^2+^, Ni^2+^, Cd^2+^, Pb^2+^, and Mg^2+^, were present in a mixed solution. It is evident from Figure 4b that the removal performance of Hg^2+^ by TpPa-1 remained above 74%. In contrast, the adsorption performance of TpPa-1 for the other six metal ions was below 10%, significantly lower than that for Hg^2+^. This indicates minimal competitive effects from coexisting metal ions, demonstrating that TpPa-1 exhibits good adsorption selectivity for Hg^2+^.

### 2.4. Possible Mechanism of TpPa-1 Adsorption

**Figure 5:**
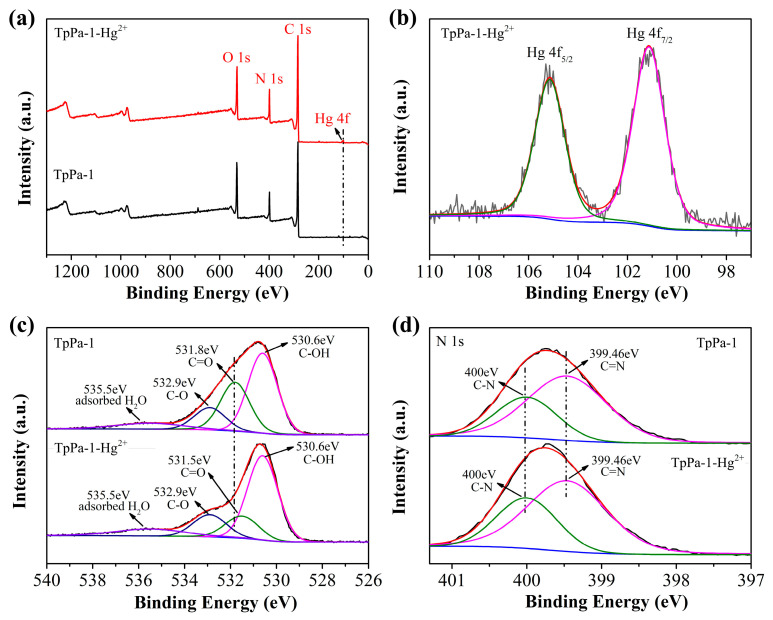
XPS survey spectrum of TpPa-1 and TpPa-1-Hg^2+^ (**a**). The high-resolution Hg 4f (**b**) spectrum for TpPa-1-Hg^2+^, and the high-resolution O 1s (**c**) and N 1s (**d**) spectra for TpPa-1 and TpPa-1-Hg^2+^.

The mechanism responsible for TpPa-1-Hg^2+^ adsorption was investigated using XPS assessments of TpPa-1 materials prior to and following Hg^2+^ adsorption. The TpPa-1 after HgCl_2_ adsorption was designated as TpPa-1-Hg^2+^. As illustrated in Figure 5a, comparison of the XPS survey spectra between TpPa-1 and TpPa-1-Hg^2+^ revealed a new Hg 4f signal in the latter, verifying successful adsorption of Hg^2+^. However, no Cl 2p peak was observed in the spectrum of TpPa-1-Hg^2+^, indicating no adsorption of Cl- to the TpPa-1 surface. In the Hg 4f spectrum of TpPa-1-Hg^2+^ (Figure 5b), two peaks were observed at binding energies equal to 101.13 and 105.14 eV, correlating, respectively, to Hg 4f_7/2_ and Hg 4f_5/2_ and indicating Hg in the +2 valence state, confirming that no redox process occurred during Hg^2+^ adsorption [15]. The O 1s spectrum of TpPa-1 (Figure 5c) showed four signals appearing at 530.6, 531.8, 532.9, and 535.5 eV related to C-OH, C=O, C-O, and chemisorbed oxygen in water or O_2_ [40]. Upon Hg^2+^ adsorption, the C=O peak shifted to 531.5 eV, suggesting interactions between Hg^2+^ and C=O moieties in TpPa-1. The N 1s spectrum obtained for TpPa-1 showed peaks at 399.46 (C=N) and 400 (C-N) eV, consistent with Schiff-base reactions between Tp and Pa-1 [32]. No shifts were observed for these two peaks within the N 1s spectrum obtained for TpPa-1-Hg^2+^ (Figure 5d). The presence of C-OH and C=N suggested that the part of TpPa-1 in the enol form has not been transformed to the keto form. These results demonstrate that Hg^2+^ adsorption likely occurs via complexation with C=O groups, where C=O competes with Cl- for coordination to Hg^2+^.

### 2.5. Reusability of TpPa-1

**Figure 6:**
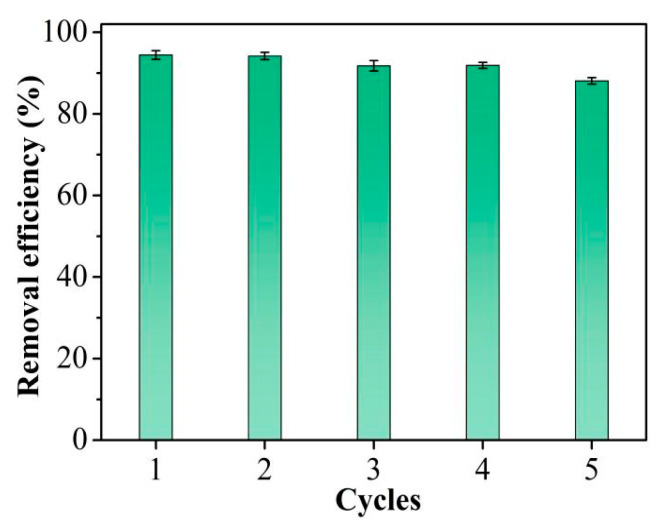
Cycle performance for Hg^2+^ removal efficiency.

Adsorbent reusability is critical in practical applications, as effective regeneration can avoid secondary pollution and reduce costs. It is evident from Figure 6 that the Hg^2+^ removal efficacy of TpPa-1 remained essentially unchanged through the four cycles. A slight decrease in removal efficiency was seen after five cycles. XRD was employed to evaluate the chemical stability of the TpPa-1 adsorbent after 5 times of reuse. The results shown in Figure S3 indicated that the crystallinity of TpPa-1 decreased slightly after 5 cycles, which may result in a decline in its removal efficacy. Nevertheless, the TpPa-1 retained at least 88% of Hg^2+^ removal efficiency after five consecutive cycles, demonstrating that this TpPa-1 adsorbent can be regenerated effectively and has potential applications for the repeated elimination of trace Hg^2+^ from water.

## 3. Materials and Methods

### 3.1. Chemicals and Instruments

Mercuric chloride (HgCl_2_, 99%) was supplied by Beijing Zhonglian Chemical Reagent Factory (Beijing, China). Lead nitrate (Pb(NO_3_)_2_, 99%) and methanol were procured from Sinopharm Chemical Reagent Co., Ltd. (Beijing, China). Tp (99%), 1,3,5-trimethylbenzene (97%), and 1,4-dioxane (99%) were supplied by Shanghai Macklin Biochemical Technology Co., Ltd. (Shanghai, China). Cobalt(II) chloride hexahydrate (CoCl_2_·6H_2_O, 99%), Nickel chloride (NiCl_2_, 98%), manganese chloride (MnCl_2_, 99%), and magnesium chloride (MgCl_2_, 99%) were obtained from Anhui Zesheng Technology Co., Ltd. (Anqing, China). Cadmium chloride (CdCl_2_, 99%), Pa-1 (99%), and Sc(OTf)_3_ (98%) were procured from Shanghai Aladdin Biochemical Technology Co., Ltd. (Shanghai, China). All chemicals were employed as received.

### 3.2. Characterization

The TpPa-1 crystal structure was assessed via a D8 Avance X-ray diffraction (XRD) system from Bruker (Karlsruhe, Germany) with Cu Kα radiation in the 2θ region extending from 2 to 40°. The surface morphology of TpPa-1 was observed using an F200 transmission electron microscope (TEM, JEOL, Tokyo, Japan) and a Regulus 8230 scanning electron microscope (SEM, Hitachi, Tokyo, Japan). The solid-state ^13^C nuclear magnetic resonance (NMR) profiles of TpPa-1 were obtained via an Avance III 400WB spectrometer from Bruker (Karlsruhe, Germany). FT-IR spectra of TpPa-1 were obtained via a Nicolet iS20 spectrometer from Thermo Fisher Scientific (Waltham, MA, USA) within the range extending from 4000 to 400 cm^−1^. An ASAP 2460 apparatus (Micromeritics, Norcross, GA, USA) was utilized to obtain N_2_ desorption/adsorption curves at a temperature equal to 77 K. Pore sizes and specific surface areas of TpPa-1 were acquired employing nonlocal density functional theory (DFT) analysis and Brunauer–Emmett–Teller (BET), respectively. The thermal stability of TpPa-1 was assessed via a Mettler Toledo (Zurich, Switzerland) TGA/DSC 3+ thermogravimetric analyzer at temperatures of 30–800 °C under a N_2_ atmosphere. An X-ray photoelectron spectroscopy (XPS) instrument (Thermo Scientific ESCALAB Xi+, East Grinstead, UK) was employed for determining the chemical distribution of TpPa-1. The zeta potential of TpPa-1 was ascertained through a Malvern Zetasizer Nano ZS90 instrument (Malvern, UK).

### 3.3. Synthesis of TpPa-1

TpPa-1 was synthesized via a Schiff-base reaction at room temperature. Specifically, Tp (0.6 mmol) and Pa (0.9 mmol) were introduced into an Erlenmeyer flask (100 mL) having 24 mL of 1,4-dioxane/mesitylene as solvent (4:1, *v*/*v*). The solution was sonicated for 15 min to completely dissolve the monomers, followed by standing for 20 min. Next, 36 µmol of Sc(OTf)_3_ was introduced, followed by sonication. The final mixture was allowed to stand for 20 min. Subsequently, the obtained reddish-brown precipitate was collected, followed by washing multiple times using methanol to eliminate unreacted reactants, and drying at 70 °C.

### 3.4. Assessment of Adsorption

For assessing the influence of pH on TpPa-1-Hg^2+^ adsorption, 3 mg of TpPa-1 were placed in a centrifuge tube (50 mL) containing 30 mL of 0.1 mg L^−1^ Hg^2+^ solutions of varying pH (3–7), adjusted utilizing 0.1 mol L^−1^ HCl or NaOH.

The adsorption capacity (*q_e_*, mg g^−1^) was determined via Equation (1):

*q_e_* = (*c*_0_ − *c*_t_) × *v*/*m* (1)

Here, *q_e_* (mg g^−1^) refers to the adsorption capacity of TpPa-1. *c*_0_ (mg mL^−1^) and *c_t_* (mg mL^−1^) refer to the concentration values of Hg^2+^ within the solution at *t* = 0 and time *t*, respectively. *v* (mL) means the volume of an adsorption solution, and *m* (g) indicates the mass of TpPa-1 adsorbent.

The isotherm adsorption studies were carried out by dispersing 3 mg of TpPa-1 with varying amounts of Hg^2+^ (0.1–20 mg L^−1^ in 30 mL, pH = 6.0), at ambient temperature for a duration equal to 12 h with shaking to achieve equilibrium between adsorption and desorption, followed by centrifugation, and filtration of the supernatants (0.22 μm PES filters). The levels of Hg^2+^ in the supernatants were measured by Atomic Fluorescence Spectroscopy (AFS). The Langmuir-Freundlich isotherm was utilized for the assessment of Hg^2+^ adsorption on TpPa-1, according to Equations (2) and (3) as follows:

(2) $$\frac{c_{e}}{q_{e}}=\frac{c_{e}}{q_{max}}+\frac{1}{K_{L}q_{max}}$$

(3) $$lnq_{e}=lnK_{F}+(1/n)lnc_{e}$$

Here, *q_max_* (mg g^−1^) represents the maximum adsorption of Hg^2+^, *c_e_* (mg L^−1^) indicates the concentration value of Hg^2+^ at equilibrium, *K_F_* and *K_L_* indicate the respective Freundlich and Langmuir isotherm constants, respectively, while 1/*n* represents TpPa-1 surface heterogeneity.

In the kinetic adsorption tests, 3 mg of TpPa-1 was mixed with 30 mL of 0.1 mg L^−1^ Hg^2+^ at a pH value equal to 6.0 and shaken at room temperature for various times (2, 5, 10, 20, 30, 60, 90, 120 min). To assess the adsorption behavior, the kinetics of Hg^2+^ adsorption on TpPa-1 were fitted through Equation (4) (pseudo-first order (PFO)) and Equation (5) (pseudo-second order (PSO)).

(4) $$ln(q_{e}-q_{t})=lnq_{e}-k_{1}t$$

(5) $$\frac{t}{q_{t}}=\frac{1}{k_{2}q_{e}^{2}}+\frac{t}{q_{e}}$$

Here, *q_t_* (mg g^−1^) and *q_e_* (mg g^−1^) represent the respective contents of Hg^2+^ adsorbed at times *t* and at equilibrium, while *k*_1_ (min^−1^) and *k*_2_ (g min^−1^ mg^−1^) are the rate constants derived from the respective PFO and PSO models.

### 3.5. Adsorption Selectivity Investigation

The selective adsorption efficacy of TpPa-1 for Hg^2+^ in water was investigated when other metal ions (Mn^2+^, Co^2+^, Ni^2+^, Pb^2+^, Cd^2+^, Mg^2+^) were present. Specifically, 3 mg of TpPa-1 was added to 30 mL of a mixed solution (pH = 6) containing Hg^2+^ and six other metal ions, where the concentration of each of these seven metal ions was 0.1 mg L^−1^. After shaking at ambient temperature for a duration equal to 12 h, the mixtures were centrifuged and filtered. The content of Hg^2+^ ions was determined by AFS, while that of the other metals was evaluated with inductively coupled plasma mass spectrometry.

### 3.6. TpPa-1 Reuse

To evaluate the reusability of TpPa-1 for Hg^2+^, 5 consecutive desorption/adsorption cycles were run. The adsorption process involved dispersing 3 mg of TpPa-1 into a solution of 0.1 mg L^−1^ Hg^2+^ (30 mL) and shaking for 3 h. After adsorption, TpPa-1 underwent centrifugation, washing with HCl (0.01 mol L^−1^) and deionized water, and oven-drying at 70 °C. The regenerated TpPa-1 was then added to a fresh Hg^2+^ solution for the second adsorption process.

## 4. Conclusions

In conclusion, TpPa-1 was effectively prepared via a one-step Schiff-base reaction at room temperature. Compared with the preparation methods of other COF materials used for Hg^2+^ removal, this approach is significantly simpler, more convenient, and time-efficient. Batch adsorption experiments combined with XPS analysis demonstrated that TpPa-1 exhibited efficient removal performance for trace-level Hg^2+^, associated with the competitive chelation and electrostatic interactions between its carbonyl groups and Hg^2+^. Moreover, the existence of six coexisting metal ions (Mn^2+^, Co^2+^, Ni^2+^, Cd^2+^, Pb^2+^, Mg^2+^) had little influence on Hg^2+^ removal efficiency, indicating the high selectivity of TpPa-1. Even following 5 consecutive desorption/adsorption cycles, TpPa-1 still maintained high efficiency in removing Hg^2+^. Overall, this study presents a facile, rapid, and mild approach for the one-step room-temperature preparation of the TpPa-1 adsorbent, which has high efficiency, good selectivity, and reusability, making it an attractive adsorbent for removing trace Hg^2+^ in practical applications.

## Data Availability

The data can be obtained from the corresponding author upon reasonable request.

## Supplementary Materials

The following supporting information can be downloaded at https://www.mdpi.com/article/10.3390/molecules30234635/s1. Figure S1: TG curve of the TpPa-1; Figure S2: The pH effect on the zeta potential of TpPa-1; Figure S3: XRD patterns of TpPa-1 (black) and the reused TpPa-1 (red) after 5 cycles; Table S1: Adsorption kinetic model parameters of TpPa-1 for Hg^2+^; Table S2: Isotherm adsorption model parameters of TpPa-1 for Hg^2+^.

## Abbreviations

The following abbreviations are used in this manuscript:

AFS	Atomic fluorescence spectroscopy
BET	Brunauer–Emmett–Teller
COFs	Covalent organic frameworks
DFT	Density functional theory
NMR	Nuclear magnetic resonance
Pa-1	*p*-Phenylenediamine
PFO	Pseudo-first order
POPs	Porous organic polymers
PSO	Pseudo-second order
Sc(OTf)_3_	Scandium(III) trifluromethanesulfonate
SEM	Scanning electron microscope
Tp	2,4,6-Triformylphloroglucinol
TEM	Transmission electron microscope
XPS	X-ray photoelectron spectroscopy
XRD	X-ray diffraction
