# Supplementary material for: Rapid, Room-Temperature Synthesis of a Porous Organic Polymer for Highly Effective Removal of Trace Hg(II) from Water
Source: Molecules. 2025 Dec 2;30(23):4635. doi: 10.3390/molecules30234635 (PMC12693406; doi:10.3390/molecules30234635)
Supplement: Supplementary file 1 [file molecules-30-04635-s001.zip › molecules-3989535-supplementary.pdf]

Supporting Information

Rapid, Room-Temperature Synthesis of a Porous Organic Polymer for Highly Effective Removal of Trace Hg(II) from Water

Shucai Gao [1], Libin Wan [1,2,*], Fayun Wang [1], Haidong Gao [1], Fanghui Zhao [1], Na Li [1], Jingjing Yao [1], Yeru Liu [1] and Hongwei Liu [1]

[1] Institute of Business Scientific, Henan Academy of Sciences, Wenhua Road #87, Zhengzhou 450003, China; gsc@hnas.ac.cn (S.G.); wangfayun262@sohu.com (F.W.); ghd@hnas.ac.cn (H.G.); fhzhao98@163.com (F.Z.); linayu1985@163.com (N.L.); yaojingjing@hnas.ac.cn (J.Y.); liuyeru@hnas.ac.cn (Y.L.); liuhongwei@hnas.ac.cn (H.L.)

[2] College of Chemistry and Molecular Engineering, Zhengzhou University, Zhengzhou 450001, China

* Correspondence: lbwan@hnas.ac.cn

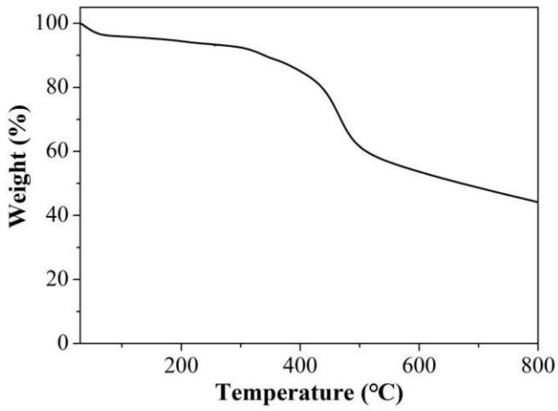

Figure S1. TG curve of the TpPa-1.

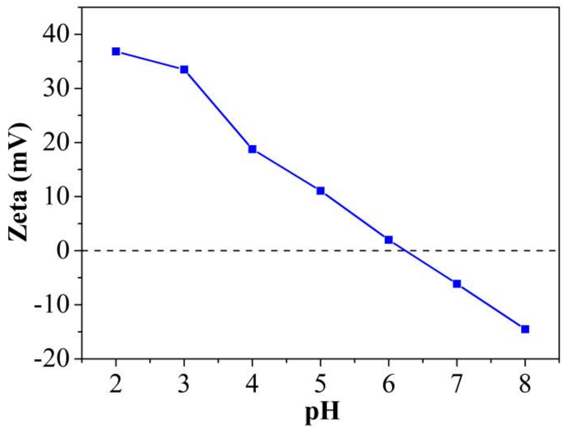

Figure S2. The pH effect on the zeta potential of TpPa-1.

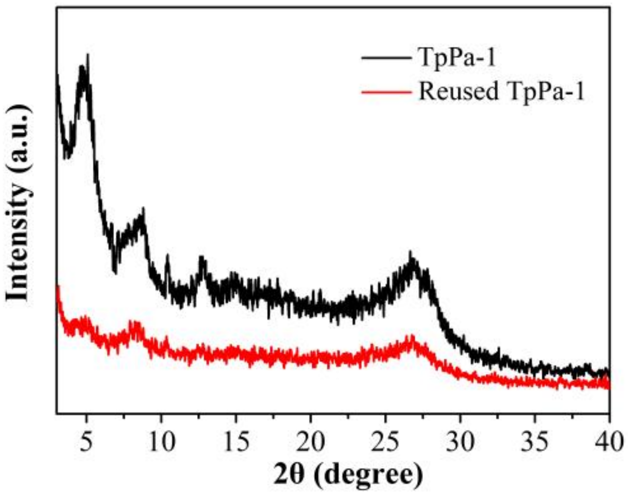

Figure S3. XRD patterns of TpPa-1 and the reused TpPa-1 after 5 cycles.

Table S1. Adsorption kinetic model parameters of TpPa-1 for Hg^{2+}.

**Pseudo first-order kinetic model**			**Pseudo second-order kinetic model**		
q_e (mg g^{-1})	k_1 (min^{-1})	R^2	q_e (mg g^{-1})	k_2 (g min^{-1} mg^{-1})	R^2
0.89	0.025	0.9877	1.03	0.039	0.9946

Table S2. Isotherm adsorption model parameters of TpPa-1 for Hg^{2+}.

**Langmuir isotherm model**			**Freundlich isotherm model**		
q_{max} (mg g^{-1})	K_L (L mg^{-1})	R^2	K_F	$1/n$	R^2
23.71	2.59	0.6985	6.23	0.436	0.9924
